# Effects of Flavonoids and Triterpene Analogues from Leaves of *Eleutherococcus sieboldianus* (Makino) Koidz. ‘Himeukogi’ in 3T3-L1 Preadipocytes

**DOI:** 10.3390/molecules22040671

**Published:** 2017-04-22

**Authors:** Atsuyoshi Nishina, Masaya Itagaki, Yuusuke Suzuki, Mamoru Koketsu, Masayuki Ninomiya, Daisuke Sato, Takashi Suzuki, Satoshi Hayakawa, Makoto Kuroda, Hirokazu Kimura

**Affiliations:** 1 College of Science and Technology, Nihon University, 1-5-1 Kandasurugadai, Chiyoda, Tokyo 101-0062, Japan; csms15006@g.nihon-u.ac.jp (M.I.); suzuki.yuusuke@nihon-u.ac.jp (Y.S.); 2 Department of Chemistry and Biomolecular Science, Faculty of Engineering, Gifu University, 1-1 Yanagido, Gifu 501-1193, Japan; koketsu@gifu-u.ac.jp (M.K.); ninomiya@gifu-u.ac.jp (M.N.); 3 Department of Biomedical Information Engineering, Graduate School of Medical Science, Yamagata University, 2-2-2 Iidanishi, Yamagata 990-9585, Japan; d_sato@yz.yamagata-u.ac.jp; 4 School of Pharmacy, Nihon University, 7-7-1 Narashinodai, Funabashi, Chiba 274-8555, Japan; suzuki.takashi85@nihon-u.ac.jp; 5 Department of Pathology and Microbiology ,School of Medicine, Nihon University, 30-1 Ohotaniguchi-kamicho, Itabashi, Tokyo 173-8610, Japan; hayakawa.satoshi@nihon-u.ac.jp; 6 National Institute of Infectious Diseases, 4-7-1 Gakuen, Musashimurayama, Tokyo 208-0011, Japan; makokuro@niid.go.jp (M.K.); kimhiro@nih.go.jp (H.K.)

**Keywords:** *Eleutherococcus sieboldianus* (Makino) Koidz., adipogenesis, lipolysis, lipogenesis, glucose transporter type 4

## Abstract

*Eleutherococcus sieboldianus* (Makino) Koidz. is a local product from the area in and around Yonezawa City in Yamagata Prefecture, Japan. It has been used as a medicinal plant for a long time. We isolated and identified four types of flavonoid glycosides [astragalin (**1**), isoquercetin (**2**), rhamnocitrin 3-*O*-glucoside (**3**), and nicotiflorin (**4**)], a triterpene [methyl hederagenin (**5**)], and three types of triterpene glycosides [δ-hederin (**6**), echinocystic acid 3-*O*-arabinoside (**7**), and cauloside B (**8**)] from the methanol extract of *E. sieboldianus*, which regulates lipid accumulation in 3T3-L1 preadipocytes. Among the compounds isolated, **2** and **8** up- and down-regulated lipid accumulation and insulin induced adipocyte differentiation in 3T3-L1 preadipocytes. Compound **2** induced up-regulation of lipid accumulation and decreased adipocyte size, while **8** down-regulated lipid accumulations without decreasing cell size. Additionally, **2** increased adipogenic proteins [peroxisome proliferator-activated receptor γ (PPARγ), CCAAT/enhancer-binding protein alpha (C/EBPα), and fatty-acid-binding protein 4 (FABP4)]. In contrast, **8** decreased the levels of all adipogenic proteins and glucose transporter type 4 (GLUT4), but increased adiponectin.

## 1. Introduction

*Eleutherococcus sieboldianus* (Makino) Koidz. (fiveleaf aralia) is a local product from the area in and around Yonezawa City in Yamagata Prefecture, Japan. *E. sieboldianus* has been used as a medicinal plant for preventing cancer and cardiovascular diseases and it is regarded as a longevity drug [1]. Additionally, *E. sieboldianus* has been used as a traditional Korean medicine (min gal pi) [2]. To date, constituents from *E. sieboldianus* have not been elucidated, except for saponins [3]. Recently, the biological activities of crude extracts of *E. sieboldianus* have been reported, including anti-oxidant, anti-diabetic, anti-obesity, and anti-microbial properties, and the lowering of blood lactate for the usage of functional food [1,4,5,6]. However, the bioactive components have not been clarified. We were interested in identifying the anti-diabetic and anti-obesity components from *E. sieboldianus,* because treating these diseases will contribute to improving human health.

Obesity is promoted by an imbalance between energy intake and output that is characterized by excessive lipid accumulation and dysfunction in adipose tissue. Additionally, being overweight and obese are recognized as major causes of death. It is estimated that 2.8 million adults die due to excess weight or obesity every year [7]. Adipocytes control energy balance and glucose homeostasis by secreting adipokines [8], and may also promote the secretion of proinflammatory cytokines such as tumor necrosis factor (TNF-α), interleukin-6 (IL-6), and interleukin-1β (IL-1β). This leads to insulin signal transduction which is down-regulated by the inhibition of insulin-stimulated tyrosine phosphorylation followed by the depression of glucose uptake [9,10,11]. Furthermore, hypertrophic adipocytes decrease the secretion of adiponectin (a hormone that regulates energy expenditure, glucose homeostasis, and insulin sensitivity) [12]. Therefore, examining the increase in adiponectin levels may prove to be an efficient method to improve insulin resistance. In support of this theory, it was reported that oral administration of adiponectin to diabetic rats improved their insulin resistance [13]. Because smaller adipocytes secrete more adiponectin and their insulin resistance may be lower, differentiation of hypertrophic adipocytes to smaller ones may be an effective strategy to improve insulin resistance and obesity [14,15].

Thiazolidinediones (TZDs), which are drugs used to treat type 2 diabetes (T2D), may improve both insulin resistance and obesity by increasing the number of small adipocytes [16]. TZDs, agonists of peroxisome proliferator-activated receptor γ (PPARγ), up-regulate PPARγ transcriptional activity, which promotes adipocyte differentiation [17,18]. TZDs produce side effects such as edema; however, they also produce therapeutic improvements in diabetes and obesity [19]. Therefore, there is a great need to develop treatments for, or methods to prevent, T2D and obesity that have fewer side effects.

Flavonoids are polyphenolic compounds that exist in various plants and are classified into flavonol, flavone, flavanone, anthocyanin, flavan-3-ols, isoflavone, and their polymers, as well as glycoside. Flavonoids have been shown to improve insulin resistance and to promote adipocyte differentiation when treated with plant polyphenols that exhibit anti-cancer, anti-inflammatory, anti-oxidative, anti-obesity, and anti-diabetic properties [20,21]. Moreover, nobiletin [22], sakuranetin [23], and magnolol [24] promote adipocyte differentiation by promoting the expression of PPARγ and CCAAT/enhancer-binding protein α (C/EBPα), which are regulators of adipocyte differentiation. Similarly, anti-adiposity effects of flavonoids have been reported [23,25,26,27].

Triterpenes are composed of various groups of natural compounds that are biosynthesized from active isoprene. Up-regulation of glucose transporter type 4 (GLUT4) expression by pachymic acid and ursolic acid [28,29,30] and the development of obesity in mice with high-fat diets using triterpene alcohol and sterol [31] have been reported.

In the present study, the effects of four types of flavonoid glycosides, three types of triterpene glycosides, and a triterpene isolated from *E. sieboldianus* on 3T3-L1 cells were evaluated, and this study found that a flavonoid glycoside and a triterpene glycoside exhibited anti-diabetic and anti-obesity effects respectively. Furthermore, the study attempted to elucidate the structure–activity relationships and mechanisms of the actions of these *E. sieboldianus* components.

## 2. Results

### 2.1. Yields, Cytotoxicity, and Regulatory Effects on Adipogenesis of the Extracts from E. sieboldianus

**Figure 1:**
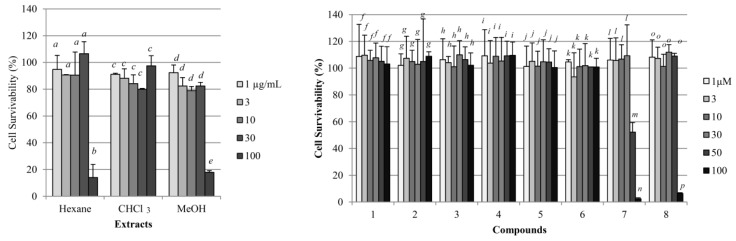
Cytotoxic effects of the three extracts and eight compounds isolated from *E. sieboldianus* in 3T3L1 cells. Data are expressed as the mean ± SD from three independent experiments. The same letters indicate that there are no differences between those groups, and different letters indicate significant differences (*p* < 0.05).

**Figure 2:**
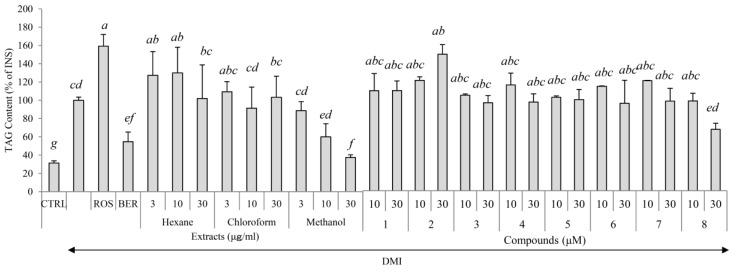
The effects of the three extracts and eight compounds isolated from *E. sieboldianus* on triglycerol levels in 3T3-L1 cells. The 3T3-L1 cells were cultured in 24-well plates and differentiated under the conditions described in the materials and methods section for each compound. Undifferentiated cells, cells with the addition of the MDI mixture (a mixture of 0.5 mM 3-isobutyl-1-methyl xanthine (M), 0.1 μM dexamethasone (D), and 2 μM insulin (I)), rosiglitazone, and berberine, are indicated by CTRL, INS, ROS, and BER, respectively. On day 8 of culturing, the medium was removed and cells were lysed using Ripa buffer. Triglycerol levels were determined by the Triglycerol E-test Wako (Wako Pure Chemical, Osaka, Japan). Data are presented as the mean ± SD from three independent experiments. The same letters indicate that there are no differences between those groups, and different letters indicate significant differences (*p* < 0.05).

Hexane extract (43 g), chloroform extract (28 g), and methanol extract (227 g) were obtained from 1846 g of the dried leaves of *E. sieboldianus*. The cytotoxicity and the regulation of intracellular triglycerol levels by these extracts are shown in Figure 1 and Figure 2. At a concentration of 30 μg/mL, the cytotoxicity of all extracts was negligible. Among the three extracts, only the methanol extract inhibited triglycerol accumulation dose dependently. Thus, the methanol extract was used to isolate the compounds that regulate adipogenesis.

### 2.2. Characterization of the Compounds Isolated

**Figure 3:**
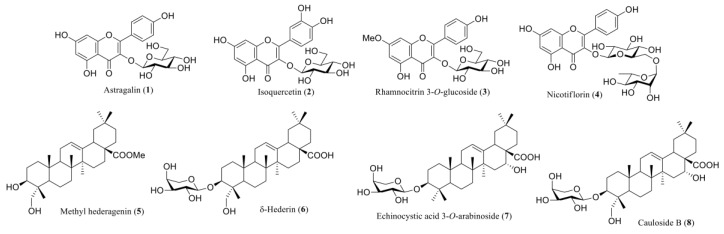
Compounds isolated from *E. sieboldianus*.

All of the compounds were identified by comparison of their spectral data with the literature. Astragalin (**1**) was previously isolated from the *Astragalus* species [32]. Compounds **2**, **3**, and **4** also displayed flavonol glycoside characteristics in NMR and were determined as isoquercetin (**2**), rhamnocitrin 3-*O*-glucoside (**3**), and nicotiflorin (**4**), respectively [33,34]. The NMR data of compounds **5**–**8** possessed the characteristics of triterpene and their structures were to be methyl hederagenin (**5**), δ-hederin (**6**), echinocystic acid 3-*O*-arabinoside (**7**), and cauloside B (**8**) (Figure 3) [35,36,37,38].

### 2.3. Cytotoxicity and Regulatory Effects on Adipogenesis of the Compounds Isolated from E. sieboldianus

We examined cell survival among 3T3-L1 cells cultured with these compounds for 8 days as described in *4.7*. (Figure 1). Although **7** and **8** induced cytotoxicity at 50 and 100 μM, respectively, compounds **1**–**6** did not induce cytotoxicity at 100 μM. Therefore, the effects on adipogenesis in the 3T3-L1 cells of each compound were evaluated at 10 and 30 μM under the conditions stated in the materials and methods section. 

**Figure 4:**
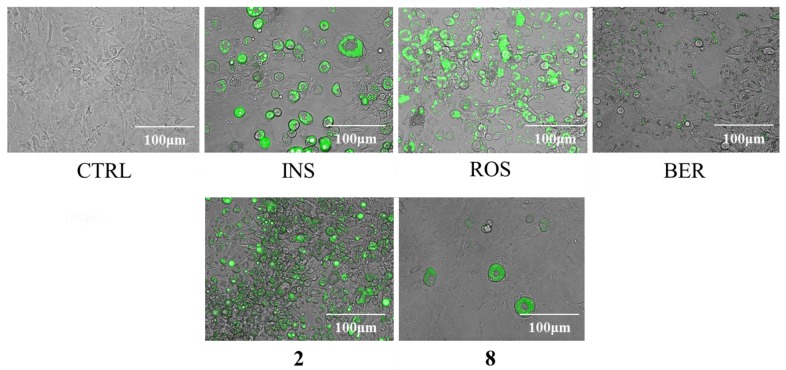
Merged phase differences and fluorescent images of differentiated 3T3-L1 cells on day 8 with reference compounds, **2**, and **8**. The 3T3-L1 cells were cultured in 24-well plates and differentiated with each compound under the conditions described in the materials and methods section. Fluorescent staining of intracellular lipids was accomplished by adding BODIPY^®^ 493/503 to the medium. Undifferentiated cells, cells with the addition of MDI mixture, rosiglitazone, and berberine are indicated by CTRL, INS, ROS, and BER respectively.

Differentiation of the 3T3-L1 cells to adipocytes was achieved within 8 days in the presence of compounds **1**–**8** at concentrations of 10 and 30 μM as described in *4.6* , and the accumulation of intracellular lipids were measured (Figure 2). When rosiglitazone (ROS) or berberine (BER) was added to the medium, with a mixture of 0.5 mM 3-isobutyl-1-methyl xanthine (M), 0.1 μM dexamethasone (D), and 2 μM insulin (I) (MDI), lipid accumulation increased by 59% and decreased by 47% respectively. Lipid accumulation was decreased by 32% when **8** (30 μM) and the MDI mixture were added to the medium compared with the addition of the MDI mixture only, but the addition of **2** (30 μM) up-regulated the levels of intracellular lipids by 43% (Figure 2). The images shown in Figure 4 indicated that the size of the 3T3-L1 cells was diminished following the addition of ROS and also when **2** and the MDI mixture were added. In contrast, the addition of **8** and the MDI mixture led to decreased intracellular lipids, but unlike ROS, miniaturization of the adipocytes did not occur.

### 2.4. The Effects of ***2*** and ***8*** on Adipogenic Proteins

**Figure 5:**
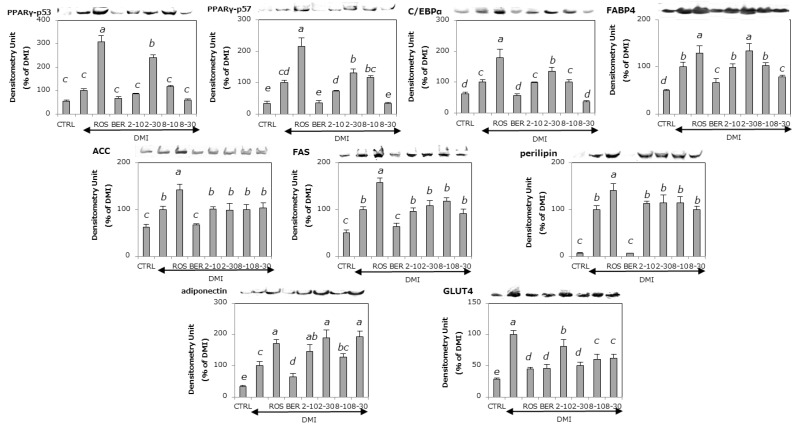
The effects of each compound on adipogenesis-related protein levels in 3T3-L1 cells during adipogenesis. The cells were differentiated under the conditions shown in Figure 4. Protein levels were measured by electroblotting. Data are presented as the mean ± SD from three independent experiments. The same letters indicate no differences between groups, and different letters indicate significant differences (*p* < 0.05).

The expression levels of the adipogenic proteins examined, namely, PPARγ, C/EBPα, and FABP4 in 3T3-L1 cells that were differentiated using MDI mixture with or without ROS, BER, **2**, or **8** are shown in Figure 5. ROS, a positive reference compound, and **2** showed that up-regulation of the expression levels of adipogenic proteins (PPARγ, C/EBPα, and FABP4) was found. In contrast, BER, a negative reference compound, and **8** down-regulated the expression of adipogenic proteins. 

### 2.5. The Effects of ***2*** and ***8*** on the Expression of Lipogenic Proteins

Acetyl-CoA carboxylase (ACC) and fatty acid synthase (FAS) are enzymes that synthesize malonyl-CoA from acetyl-CoA and fatty acids and from acetyl-CoA and malonyl-CoA respectively [39]. The effects of **2** and **8** on the levels of lipogenic proteins are shown in Figure 5. ROS showed up-regulation of the expression of ACC and FAS. BER down-regulated the expression of ACC and FAS. In contrast, adding **2** or **8** did not change the expression of ACC and FAS.

### 2.6. The Effects of ***2*** and ***8*** on the Expression of a Lipolytic Protein

Perilipin coats lipid droplets in adipocytes and induces lipolysis [40,41]. The effects of **2** and **8** on the levels of perilipin are shown in Figure 5. Similar to the effects on the levels of lipogenic proteins, ROS up-regulates and BER down-regulates the expression of perilipin. Adding **2** or **8** did not change perilipin levels.

### 2.7. The Effects of ***2*** and ***8*** on the Expression of Adiponectin

Adiponectin is a hormone controlling energy expenditure, glucose homeostasis, and insulin resistance [9]. Insulin resistance is improved by increasing adiponectin [10]. In addition, it has been reported that insulin resistance in diabetic mice was improved by oral administration of adiponectin [12]. The effects of **2** and **8** on the levels of adiponectin are shown in Figure 5. ROS, Compound **2**, and **8** up-regulated the expression levels of adiponectin dose dependently. In contrast, the addition of BER down-regulated the expression levels of adiponectin.

### 2.8. The Effects of ***2*** and ***8*** on the Expression of GLUT4

GLUT4 accumulates in intracellular organelles (endosomes) in an environment without insulin stimulation. Once insulin binds to its receptor in the cell membrane, phosphoinositide 3-kinase (PI3K), Akt, and other downstream components are activated in turn, followed by the translocation of GLUT4 to the cell membrane. GLUT4 in the cell membrane takes glucose up from the blood [42]. In addition, the translocation of GLUT4 to the cell membrane is closely related to adipogenesis [43]. The expression of GLUT4 in 3T3-L1 cells that were differentiated under the same conditions as mentioned in Figure 4 is shown in Figure 5. ROS and BER up- and down-regulated GLUT4 protein levels respectively. Compounds **2** and **8** showed dose-dependent up-regulation of GLUT4 protein levels.

## 3. Discussion

To examine the effect of *E. sieboldianus* and some of its compounds on 3T3-L1 preadipocytes, we isolated and identified four types of flavonoid glycosides [astragalin (**1**), isoquercetin (**2**), rhamnocitrin 3-*O*-glucoside (**3**), and nicotiflorin (**4**)], a triterpene [methyl hederagenin (**5**)], and three types of triterpene glycosides [δ-hederin (**6**), echinocystic acid 3-*O*-arabinoside (**7**), and cauloside B (**8**)] from the methanol extract of *E. sieboldianus*, which regulates lipid accumulation and adipogenesis in 3T3-L1 preadipocytes. 

Compound **1**–**8** were isolated from *Morus alba* [44], *Astragalus hamosus* [45], *Astragalus complanatus* [46], *Brickellia cavanillesii* [47], *Achyranthes bidentate* [48], *Hedera helix* [49], *Albizia inundata* [50], and *Symphytum officinale* [51], respectively, though these compounds have not been described previously for *E. sieboldianus*.

Among the compounds isolated, **2** and **8** up- and down-regulated, respectively, lipid accumulation in 3T3-L1 preadipocytes. Compound **2** induced up-regulation of lipid accumulation and decreased adipocyte size, while **8** down-regulated lipid accumulation without decreasing cell size (Figure 3). Additionally, **2** increased adipogenic proteins (PPARγ, C/EBPα, and FABP4) in a dose dependent manner (Figure 5). In contrast, **8** decreased the levels of all adipogenic proteins and GLUT4, but increased adiponectin. Because it showed promotion of lipid accumulation equivalent to ROS (Figure 2), **2** may be one of the main active components from *E. sieboldianus*. On the other hand, the components other than **8** (with its inhibition of lipid accumulation) may be responsible for the use of *E. sieboldianus*, because the effect of **8** was weaker than that of the methanol extract.

Though saponins and flavonoid glycoside (nicotiflorin) are known to be constituents of *E. sieboldianus* [3], there have been no reports on compounds except nicotiflorin that have been identified from *E. sieboldianus.*

Only **2** up-regulated lipid accumulation among the four types of flavonoid glycosides, which indicates that the number and position of the hydroxyl groups binding to the B ring of the flavonol skeleton controls this effect. It has been shown that flavonoids, especially flavonol, flavan-3-ol and anthocyanins decrease glycemia, which is followed by an improvement in the secretion and sensitivity of insulin [52]. Furthermore, it has also been reported that anthocyanins improve glucose metabolism, insulin resistance, and β cell dysfunction via regulation of GLUT4 [53,54]. In addition, we showed that the presence or absence of a hydroxyl group binding to the C23 position of triterpene controls the ability to regulate lipid accumulation because the regulation of lipid accumulation significantly differed between **7** and **8**. The relationship between the chemical structure of triterpenoids and anti-diabetic effects is unclear, however, it has been reported that triterpene analogues, such as ginsenosides, regulate adipogenesis [55,56,57,58]. Further studies, including the findings obtained in the present study, may be needed to clarify the mechanisms of action, even though there are many reports concerning anti-diabetic or anti-obesity effects of flavonoid and triterpene analogues.

Increases in hypertrophic adipocytes regulate the secretion of adiponectin (a hormone regulating energy expenditure), glucose homeostasis, and insulin sensitivity. In contrast, because smaller adipocytes secrete more adiponectin and improve sensitivity to insulin, decreasing the size of hypertrophic adipocytes, followed by increasing the number of smaller adipocytes, is considered to be one strategy to treat diabetes [13]. 

In the present study, compound **2** and ROS decreased the size of adipocytes produced by insulin stimulation, as shown in Figure 4. In contrast, unlike ROS (an agonist of PPARγ) [59], **2** decreased the size of adipocytes but was irrelevant to the expression of lipogenic and lipolytic proteins (Figure 5); thus, **2** may show antidiabetic effects through a different mechanism of action than thiazolidinediones (TZDs) such as ROS, the drugs that are widely used to treat diabetes.

Furthermore, compound **8** showed a dose-dependent anti-obesity effect, as seen in Figure 3. The anti-obesity effect of **8** was indicated because **8** down-regulated the levels of three types of adipogenic proteins and GLUT 4 as is shown in Figure 5. Because **8** did not decrease the size of adipocytes but did up-regulate the secretion of adiponectin, **8** may induce anti-obesity effects by improving the insulin resistance of adipocytes.

PPARγ was identified as an indispensable transcription factor that regulates adiposity through analysis of gene-deficient mice [60]. Moreover, C/EBPα is a transcription factor regulating the acquisition of insulin sensitivity [61]. In contrast, FABP4, which is expressed in adipocytes and macrophages, is strongly related to inflammation and lipid homeostasis in cells [62]. In the present study, compounds **2** and **8** up- or down-regulated adipogenic proteins and they up-regulated adiponectin and down-regulated GLUT4. In contrast, **2** and **8** were irrelevant to the expression of lipogenic and lipolytic proteins (Figure 5). This suggests that the regulation of adipogenesis by **2** and **8** were not related to the expression levels of GLUT4. Furthermore, the reason for the up-regulation of the levels of adiponectin without downsizing of adipocyte by DMI should be clarified in the future.

Anti-obesity compounds are therapeutic agents that can reduce body weight by decreasing the consumption or absorption of food, and/or increasing energy expenditure [63]. Because the causes of obesity are various, it is necessary to use various treatments, according to the characteristics of the patient. In the present study, effects that decrease lipid accumulation by stimulation using compound **8** are recognized as one type of anti-obesity treatment. The *in vivo* trials are also necessary to practicality access the anti-adiposity activity of compound **8**.

In the present study, the effect on the glucose uptake of the cells treated with compound **2** and **8** was deduced by the evaluation of expression levels of GLUT4 in the cytoplasm. It was clarified that both compound **2** and **8** down-regulated the expression of GLUT4. On the other hand, it is necessary to evaluate the GLUT4 levels in the cell membrane and the amount of uptake of glucose to confirm the effects of compound **2** and **8** in more detail. Though TZDs like ROS are widely used to treat diabetes, they have side effects such as edema. Because *E. sieboldianus* contains both anti-diabetic and anti-obesity compounds such as **2** and **8**, and because extracts from *E. sieboldianus* improve insulin resistance in type 2 diabetic mice [4,6], *E. sieboldianus* may be a promising natural anti-diabetic or anti-obesity agent.

Because anti-diabetic compounds stabilize and control blood glucose levels [64], the evaluation of the biological effects of compound **2** in people with diabetes or in animal models of this disease from the viewpoint of blood glucose levels is necessary to confirm the practical effects of compound **2** in detail. 

## 4. Materials and Methods

### 4.1. Chemicals and Reagents

Rosiglitazone [ROS; a type of Thiazolidinedione (TZD)] and Dulbecco’s modified Eagle’s medium (DMEM) were purchased from Wako Pure Chemical (Osaka, Japan). Fetal bovine serum (FBS), calf serum, and BODIPY^®^ 493/503 were purchased from Life Technologies (Carlsbad, CA, USA). Insulin (INS), dexamethazone (DEX), 3-isobutyl-1-methylxanthine (IBMX), and berberine (BER) were obtained from Sigma-Aldrich (St. Louis, MO, USA).

### 4.2. Solvent Fractionation

Raw leaves of *Eleutherococcus sieboldianus* (Araliaceae) was purchased commercially in Yamagata Prefecture in Japan and identified by Prof. Nishina. The voucher specimen has been deposited at the College of Science and Technology, Nihon University. Dried *E. sieboldianus* powder (1846 g) was immersed in hexane (500 mL) for 24 h at room temperature. The solvent containing the extracts was filtrated through a filter paper (5C; Whatman, Brentford, UK) and the filtrate was evaporated to dryness to prepare hexane extract. The residue was then stirred in chloroform (500 mL) at room temperature for 24 h, filtrated, and the filtrate was dried in vacuo to prepare chloroform extract. Then, methanol extract was obtained in the same manner.

### 4.3. Isolation of Active Constituents

The methanol extract (227 g) was partitioned with *n*-hexane, ethyl acetate, and *n*-butanol in that order and each fraction of *n*-hexane (25 g), ethyl acetate (37 g), and *n*-butanol (78 g) was obtained. The ethyl acetate fraction (37 g) was divided by silica gel column chromatography (CC) eluted with CHCl_3_/MeOH (1/0 to 5/2; *v*/*v*), to give seven fractions (Fr. 1 to Fr. 7). Repeated silica gel CC of Fr. 2 (2.0 g) eluting with CHCl_3_/MeOH = 1/0 to 20/1 afforded compound **5** (65 mg). Fr. 3 (9 g) was separated by silica gel CC (CHCl_3_/MeOH = 1/0 to 20/1) to give five subfractions (subFr. 3-1 to subFr. 3-5). Purification of subFr. 3-2 (1.02 g) using Sephadex LH-20 CC and washing with acetone gave compound **7** (890 mg). SubFr. 3-3 (1.02 g) was washed with acetone and then filtered the white solid, to afford the additional amount of compound **7** (935 mg). Several washing of subFr. 3-4 (3.08 g) with acetone yielded compound **6** (900 mg). Continuous separation of subFr. 3-5 (1.29 g) by Sephadex LH-20 and silica gel (CHCl_3_/MeOH = 10/1) CC gave compound **8** (42 mg). Repeated Sephadex LH-20 and Octa Decyl Silyl (ODS) (MeOH) CC of Fr. 4 (2.0 g) afforded compound **1** (320 mg) and subFr. 4-2-1-2 (209 mg). Further purification of subFr. 4-2-1-2 using RP-HPLC eluted with 22% CH_3_CN/H_2_O yielded compound **2** (165 mg). Fr. 5 (345 mg) was applied to Sephadex LH-20 CC eluting MeOH to yield compound **3** (9 mg). Fr. 6 (649 mg) was purified by Sephadex LH-20 CC with MeOH elution to afford compound **4** (270 mg).

Purity of compound **1**–**9** was confirmed as more than 95% by measurement of ^1^H-NMR spectra with dimethyl sulfone as the internal standard [65].

### 4.4. Analytical Instrument of Active Components

^1^H (400 MHz) and ^13^C (100 MHz) NMR spectra were recorded with a JEOL ECX 400 spectrometer with tetramethylsilane as an internal standard. MS spectra were obtained using a JEOL JMS-700/GI spectrometer and the Waters UPLC-MS system (Aquity UPLC XevoQTof). IR spectra were recorded on a JASCO FT/IR-460 Plus spectrophotometer.

### 4.5. Spectral Data of Isolated Components

*Astragalin* (**1**): Yellow powder. IR (film): 3372, 1657, 1607, 1363, 1115, 1065 cm^−1^. FABMS: *m*/*z* 449 [M + H]^+^. HRFABMS: *m*/*z* 449.1085 [M + H]^+^ (calcd. for C_21_H_21_O_11_, 449.1084). ^1^H-NMR (400 MHz, DMSO-*d*_6_): δ 8.04 (2H, d, *J* = 9.2 Hz, H-2′ and H-6′), 6.89 (2H, d, *J* = 9.2 Hz, H-3′ and H-5′), 6.44 (1H, d, *J* = 1.8 Hz, H-8), 6.21 (1H, d, *J* = 1.8 Hz, H-6), 5.46 (1H, d, *J* = 7.4 Hz, H-1″), 3.57 (1H, dd, *J* = 11.9 and 5.0 Hz, H-6α″), 3.38–3.31 (1H, m, H-6β″), 3.28–3.15 (2H, m, H-2″ and H-3″), 3.11–3.05 (2H, m, H-4″ and H-5″). ^13^C-NMR (100 MHz, DMSO-*d*_6_): δ 178.0, 164.7, 161.7, 160.5, 156.9, 156.8, 133.7, 131.4, 121.4, 115.6, 104.5, 101.4, 99.2, 94.2, 78.0, 76.9, 74.7, 70.4, 61.4.

*Isoquercetin* (**2**): Yellow powder. IR (film): 3391, 1659, 1606, 1367, 1065 cm^−1^. FABMS: *m*/*z* 465 [M + H]^+^, 303 [M + H – Glc]^+^. HRFABMS: *m*/*z* 465.1051 [M + H]^+^ (calcd. for C_21_H_21_O_12_, 465.1033). ^1^H-NMR (400 MHz, CD_3_OD): δ 7.72 (1H, s, H-2′), 7.57 (1H, d, *J* = 7.3 Hz, H-5′), 6.86 (1H, d, *J* = 7.3 Hz, H-6′), 6.38 (1H, s, H-8), 6.19 (1H, s, H-6), 5.25 (1H, d, *J* = 7.4 Hz, H-1″), 3.72 (1H, d, *J* = 11.4 Hz, H-6α″), 3.58 (1H, dd, *J* = 11.4 and 5.0 Hz, H-6β″), 3.52–3.41 (1H, m, H-2″), 3.39–3.22 (3H, m, H-3″, H-4″, and H-5″). ^13^C-NMR (100 MHz, CD_3_OD): δ 178.1, 164.7, 161.7, 157.7, 157.1, 148.5, 144.5, 134.3, 121.9, 121.7, 116.2, 114.6, 104.3, 103.0, 98.6, 93.4, 77.0, 76.8, 74.4, 69.9, 61.2.

*Rhamnocitrin 3-O-glucoside* (**3**): Yellow powder. IR (film): 3391, 1651, 1598, 1381, 1067 cm^−1^. FABMS: *m*/*z* 485 [M + Na]^+^, 463 [M + H]^+^. HRFABMS: *m*/*z* 463.1260 [M + H]^+^ (calcd. for C_22_H_23_O_11_, 463.1240). ^1^H-NMR (400 MHz, CD_3_OD): δ 8.08 (2H, d, *J* = 8.7 Hz, H-2′ and H-6′), 6.88 (2H, d, *J* = 8.7 Hz, H-3′ and H-5′), 6.59 (1H, d, *J* = 2.3 Hz, H-8), 6.32 (1H, d, *J* = 2.3 Hz, H-6), 5.29 (1H, d, *J* = 7.8 Hz, H-1″), 3.88 (3H, s, 7-OMe), 3.69 (1H, dd, *J* = 11.9 and 2.3 Hz, H-6α″), 3.52 (1H, dd, *J* = 11.9 and 5.5 Hz, H-6β″), 3.49–3.40 (2H, m, H-2″ and H-3″), 3.35–3.19 (3H, m, H-3″, H-4″, and H-5″). ^13^C-NMR (100 MHz, CD_3_OD): δ 178.3, 166.0, 161.5, 160.3, 158.0, 157.1, 134.3, 131.0, 121.4, 114.8, 105.3, 102.6, 97.7, 91.8, 77.1, 76.7, 74.4, 70.0, 61.3, 55.2.

*Nicotiflorin* (**4**): Yellow powder. IR (film): 3372, 1656, 1606, 1361, 1065 cm^−1^. FABMS: *m*/*z* 595 [M + H]^+^. HRFABMS: *m*/*z* 595.1660 [M + H]^+^ (calcd. for C_27_H_31_O_15_, 595.1663). ^1^H-NMR (400 MHz, DMSO-*d*_6_): δ 7.99 (2H, d, *J* = 9.2 Hz, H-2′ and H-6′), 6.88 (2H, d, *J* = 9.2 Hz, H-3′ and H-5′), 6.42 (1H, d, *J* = 2.3 Hz, H-8), 6.21 (1H, d, *J* = 2.3 Hz, H-6), 5.31 (1H, d, *J* = 7.8 Hz, H-1″), 4.38 (1H, d, *J* = 1.4 Hz, H-1′′′), 3.69 (1H, d, *J* = 10.1 Hz, H-6α″), 3.38–3.00 (9H, m, H-2″–H-6β″ and H-2′′′–H-5′′′), 0.98 (3H, d, *J* = 6.4 Hz, H-6′′′). ^13^C-NMR (100 MHz, DMSO-*d*_6_): δ 177.9, 164.6, 161.7, 160.4, 157.4, 157.0, 133.7, 131.4, 121.4, 115.6, 104.5, 101.8, 101.3, 99.2, 94.3, 76.8, 76.3, 74.7, 72.3, 71.1, 70.9, 70.4, 68.8, 67.4, 18.2.

*Methyl hederagenin* (**5**): White powder. IR (film): 3393, 1701, 1598, 1107 cm^−1^. FABMS: *m*/*z* 487 [M + H]^+^, 469 [M + H – H_2_O]^+^. HRFABMS: *m*/*z* 469.3663 [M + H – H_2_O]^+^ (calcd. for C_31_H_49_O_3_, 469.3682). HRESITOFMS: *m*/*z* 509.3588 [M + Na]^+^ (calcd. for C_31_H_50_O_4_Na, 509.3607). ^1^H-NMR (400 MHz, CD_3_OD): δ 5.25 (1H, t, *J* = 3.6 Hz, H-12), 3.62 (3H, s, COOMe), 3.61–3.56 (1H, m, H-3), 3.52 (1H, d, *J* = 11.0 Hz, H-23b), 3.28 (1H, d, *J* = 11.0 Hz, H-23a), 2.86 (1H, dd, *J* = 14.2 and 4.6 Hz, H-18), 2.09–1.99 (1H, m, H-11b), 1.90 (1H, dd, *J* = 9.2 and 3.7 Hz, H-11a), 1.74–0.87 (22H, m, aglycone), 1.16 (3H, s, H-27), 0.97 (3H, s, H-25), 0.93 (3H, s, H-30), 0.91 (3H, s, H-29), 0.74 (3H, s, H-26), 0.70 (3H, s, H-24). ^13^C-NMR (100 MHz, CD_3_OD): δ 178.7, 143.7, 122.5, 72.5, 66.0, 50.9, 47.6, 46.8, 45.7, 41.9, 41.5, 41.4, 39.2, 38.1, 36.6, 33.4, 32.3, 32.2, 32.0, 30.3, 27.4, 26.1, 25.2, 23.2, 22.7, 22.6, 17.8, 16.3, 14.9, 11.4.

*δ-Hederin* (**6**): White powder. IR (film): 3392, 1690, 1599, 1068 cm^−1^. FABMS: *m*/*z* 605 [M + H]^+^, 472 [M + H – Ara]^+^, 455 [M + H – H_2_O – Ara]^+^. HEESITOFMS: *m*/*z* 627.3868 [M + Na]^+^ (calcd. for C_35_H_56_O_8_Na, 627.3873). ^1^H-NMR (400 MHz, DMSO-*d*_6_): δ 5.16 (1H, br s, H-12), 4.18 (1H, d, *J* = 6.9 Hz, H-1′), 3.65 (1H, dd, *J* = 12.4 and 3.2 Hz, H-5β′), 3.60 (1H, br s, H-4′), 3.49 (1H, dd, *J* = 11.4 and 4.1 Hz, H-3), 3.44–3.28 (4H, m, H-2′, H-3′, H-5α′, and H-23b), 3.08 (1H, d, *J* = 10.5 Hz, H-23a), 2.74 (1H, dd, *J* = 14.2 and 4.1 Hz, H-18), 1.98–0.80 (27H, m, aglycone), 1.10 (3H, s, H-27), 0.89 (3H, s, H-25), 0.87 (6H, s, H-29 and H-30), 0.71 (3H, s, H-26), 0.59 (3H, s, H-24). ^13^C-NMR (100 MHz, DMSO-*d*_6_): δ 179.1, 144.4, 122.1, 105.4, 80.3, 73.2, 71.6, 68.2, 65.6, 63.2, 47.6, 46.6, 46.2, 46.0, 42.9, 41.9, 41.4, 39.4, 38.5, 36.5, 33.8, 33.4, 32.6, 32.5, 30.9, 27.7, 26.1, 25.7, 23.9, 23.5, 23.1, 17.7, 17.4, 16.1, 13.5.

*Echinocystic acid 3-O-arabinoside* (**7**): White powder. IR (film): 3392, 1687, 1601, 1069 cm^−1^. FABMS: *m*/*z* 605 [M + H]^+^, 455 [M + H – H_2_O – Ara]^+^. HRESITOFMS: *m*/*z* 627.3877 [M + Na]^+^ (calcd. for C_35_H_56_O_8_Na, 627.3873). ^1^H-NMR (400 MHz, CD_3_OD): δ 5.29 (1H, br s, H-12), 4.46 (1H, br s, H-16), 4.27 (1H, d, *J* = 6.9 Hz, H-1′), 3.82 (1H, dd, *J* = 15.6 and 3.2 Hz, H-5β′), 3.82–3.77 (1H, m, H-4′), 3.60–3.49 (3H, m, H-2′, H-3′, and H-5α′), 3.14 (1H, dd, *J* = 11.9 and 4.6 Hz, H-3), 3.00 (1H, dd, *J* = 14.2 and 4.1 Hz, H-18), 2.29 (1H, t, *J* = 13.3 Hz, H-11b), 2.01–0.75 (24H, m, aglycone), 1.38 (3H, s, H-27), 1.04 (3H, s, H-23), 0.97 (3H, s, H-30), 0.96 (3H, s, H-25), 0.88 (3H, s, H-29), 0.84 (3H, s, H-24), 0.79 (3H, s, H-26). ^13^C-NMR (100 MHz, CD_3_OD): δ 179.8, 143.7, 122.1, 105.8, 89.4, 73.9, 73.0, 71.5, 68.2, 65.0, 55.8, 48.3, 46.8, 46.4, 41.3, 40.8, 39.3, 38.9, 38.5, 36.6, 35.2, 34.9, 33.0, 32.1, 31.4, 30.1, 27.3, 26.0, 25.7, 23.6, 23.2, 18.0, 16.5, 15.7, 14.8.

*Cauloside B* (**8**): White powder. HRESITOFMS: *m*/*z* 643.3825 [M + Na]^+^ (calcd. for C_35_H_56_O_9_Na, 643.3822). IR (film): 3391, 1699, 1599, 1068 cm^−1^. ^1^H-NMR (400 MHz, CD_3_OD): δ 5.29 (1H, br s, H-12), 4.45 (1H, br s, H-16), 4.32 (1H, d, *J* = 6.9 Hz, H-1′), 3.83 (1H, dd, *J* = 12.8 and 2.8 Hz, H-5β′), 3.79 (1H, br s, H-4′), 3.64–3.48 (5H, m, H-2′, H-3, H-3′, H-5α′, and H-23b), 3.28 (1H, d, *J* = 10.1 Hz, H-23a), 3.00 (1H, dd, *J* = 14.7 and 4.1 Hz, H-18), 2.28 (1H, t, *J* = 13.8 Hz, H-11b), 1.99–0.85 (25H, m, aglycone), 1.39 (3H, s, H-27), 0.98 (3H, s, H-25), 0.97 (3H, s, H-30), 0.88 (3H, s, H-29), 0.80 (3H, s, H-26), 0.71 (3H, s, H-24). ^13^C-NMR (100 MHz, CD_3_OD): δ 180.2, 143.8, 122.0, 105.0, 82.0, 74.0, 73.2, 71.6, 68.4, 65.5, 47.0, 46.8, 46.4, 42.5, 41.4, 40.8, 39.3, 38.2, 36.4, 35.2, 34.8, 32.4, 32.1, 31.3, 30.0, 26.0, 25.0, 23.6, 23.2, 17.5, 16.5, 15.2, 12.1.

### 4.6. Cell Culture

Murine 3T3-L1 preadipocytes were propagated in DMEM supplemented with 10% calf serum until 80% confluence (day 0) and the medium was replaced with DMEM containing 10% FBS and MDI mixture, with or without one of the test compounds. After 48 h (day 2), the medium was replaced with DMEM containing 10% FBS and 2 μM insulin. After 48 h (day 4), the medium was replaced with DMEM containing 10% FBS. Thereafter, the medium was exchanged every other day [66]. 0.1 μM or 2.7 nM of ROS or BER was used as a positive or negative reference compound respectively. Cells were maintained in a humidified atmosphere of 5% CO_2_ at 37 °C

### 4.7. Cell Toxicity Assay

The 3T3-L1 cells were seeded in 96-well plate with DMEM supplemented with 10% calf serum until 80% confluence and the medium was replaced with DMEM containing a 10% FBS with or without one of a test compound. Thereafter, we exchanged the medium without test compounds on every other day. Cytotoxicity was measured by the use of Cell Counting Kit-8 (Dojindo, Kumamoto, Japan) according to the instructions of the manufacturer. Absorbance was measured at 450 nm by using a Sunrise Absorbance Reader (Tecan, Männedorf, Switzerland).

### 4.8. Measurement of Intracellular Triglycerol Level

Intracellular triglycerol levels in 3T3-L1 cells at day 8 were measured by using of E-test WAKO Triglyceride Kit (Wako Pure Chemical) according to the instructions of the manufacturer. 100 ng/mL of BODIPY 493/503 were added to culture medium followed by incubation of 10 min. Images were taken by a fluorescent cell imager (Floid Cell Imaging Solution; Life Technologies) [66].

### 4.9. Detection of Proteins

Differentiated (day 8) 3T3-L1 cells in 6-well plates were placed on ice and each well was washed with PBS, and subsequently lysed with 150 μL of 20 mM Tris-HCl buffer (pH 8.0) containing 150 mM NaCl, 2 mM EDTA, 1% Nonidet P-40 (*w*/*v*), 1% sodium deoxycholate (*w*/*v*), 0.1% sodium dodecyl sulfate (*w*/*v*), 50 mM NaF, 0.1% aprotinin (*w*/*v*), 0.1% leupeptin (*w*/*v*), 1 mM Na_3_VO_4_, and 1 mM phenylmethylsulphonylfluoride (PMSF). Cell lysates were collected using a cell scraper and centrifuged at 15,000× *g* for 30 min at 4 °C. The supernatant was collected and the overall protein concentration was determined using a Protein Assay Reagent Kit (Cytoskeleton, Denver, CO, USA) with bovine serum albumin (BSA) as the standard. 

Supernatant fluids containing proteins were mixed with a lithium dodecyl sulfate (LDS) sample buffer (Invitrogen Corp, Carlsbad, CA, USA) and incubated for 5 min at 80 °C. Samples containing proteins (20 μg) were loaded in each lane followed by separation on SDS-polyacrylamide gel electrophoresis, and the proteins in gels were electroblotted onto polyvinylidene fluoride (PVDF) filters (Hybond-P, 0.2 μm; GE Healthcare, Little Chalfont, UK). Immunoblotting analysis was performed by using monoclonal antibodies against glyceraldehyde-3-phosphate dehydrogenase (GAPDH) (37 kDa), C/EBPα (42 kDa), FABP4 (15 kDa), PPARγ (53 and 57 kDa), acetyl-CoA carboxylase (ACC) (280 kDa), fatty acid synthase (273 kDa), perilipin (62 kDa), adioponectin (27 kDa), and GLUT4 (50 kDa) (Cell Signaling Technology, Lake Placid, NY, USA) as the primary antibodies, followed by a reaction with horseradish peroxidase-conjugated anti-rabbit IgG antibodies from Sigma-Aldrich (St. Louis, MO, USA) as the secondary antibody. Primary and secondary antibodies were diluted 1000 or 3000 times for use, respectively. The blots were developed by the enhanced chemiluminescence method (Western Lightning ECL Pro; Perkin Elmer, Waltham, MA, USA) [67].

### 4.10. Statistical Analysis

The results were expressed as mean ± standard deviation (SD). The significant difference between the groups compared was determined using analysis of variance (ANOVA) followed by the Tukey test.

## 5. Conclusions

We isolated and identified four types of flavonoid glycosides [astragalin (**1**), isoquercetin (**2**), rhamnocitrin 3-O-glucoside (**3**), and nicotiflorin (**4**)], a triterpene [methyl hederagenin (**5**)], and three types of triterpene glycosides [δ-hederin (**6**), echinocystic acid 3-*O*-arabinoside (**7**), and cauloside B (**8**)] from the methanol extract of *E. sieboldianus*, which regulates lipid accumulation and adipogenesis in 3T3-L1 preadipocytes. Among the compounds isolated, **2** and **8** up- and down-regulated lipid accumulation in 3T3-L1 preadipocytes. Compound **2** induced up-regulation of lipid accumulation and decreased adipocyte size, while **8** down-regulated lipid accumulations without decreasing cell size. Additionally, **2** increased adipogenic proteins in a dose dependent manner. In contrast, **8** decreased the levels of all adipogenic proteins and GLUT4, but increased adiponectin.
